# Spontaneously reported persistent symptoms related to coronavirus disease 2019 one year after hospital discharge: A retrospective cohort single-center study

**DOI:** 10.1007/s00482-022-00626-0

**Published:** 2022-02-25

**Authors:** David Zuschlag, Daniel Grandt, Florian Custodis, Christian Braun, Winfried Häuser

**Affiliations:** 1 Innere Medizin 1, Klinikum Saarbrücken, Winterberg 1, 66119 Saarbrücken, Germany; 2 Innere Medizin 2, Klinikum Saarbrücken, Saarbrücken, Germany; 3 Zentrale Notaufnahme, Klinikum Saarbrücken, Saarbrücken, Germany; 4 Klinikum rechts der Isar, Klinik für Psychosomatische Medizin und Psychotherapie, Technische Universität München, München, Germany

**Keywords:** COVID-19, False attribution, Chronic pain, Post COVID-19 condition, Fatigue, COVID-19, Fehlattribution, Chronische Schmerzen, Post COVID-19, Müdigkeit

## Abstract

**Background:**

There are no outcome studies for coronavirus disease 2019 (COVID-19) survivors one year after hospital discharge in Germany.

**Methods:**

This retrospective cohort study included all patients with polymerase chain reaction (PCR)-confirmed severe acute respiratory syndrome coronavirus 2 (SARS-CoV-2) hospitalized in the departments of internal medicine of the Klinikum Saarbrücken, a tertiary care hospital, between March 15 and December 31, 2020. A telephone interview with survivors was conducted at least 12 months after discharge. The interview was initiated with an open-ended question whether the patient had fully recovered from the disease. In the event of a subjective incomplete recovery, the patient was prompted to report any continuous or frequent symptoms that had not occurred prior to COVID-19. Finally, independent of the open-ended question response, all patients were asked closed questions which addressed new symptom onset of persistent fatigue, cognitive dysfunction, headache, muscle and joint pain following COVID-19.

**Results:**

In all, 235 survivors were contacted and 162 could be included in the analysis. In 55 of 162 interviews (34.0%) at least one persistent COVID-19 symptom (PCS) was spontaneously reported. Four of 55 survivors with PCS reported five additional symptoms on the closed questions. One survivor, who responded positively to the open-ended question, reported new onset PCS in response to the closed questions. Physical fatigue (24.7%), cognitive dysfunction (14.8%), shortness of breath (8.6%), muscle and joint pain (6.8%) and headache (6.2%) were the most frequently reported PCS.

**Conclusions:**

Despite an interview technique aimed to reduce attribution bias by patients, one third of COVID-19 inpatient survivors report PCS one year after hospitalization.

The complete article is written in English.

## Abstract (de)

**Hintergrund:**

In Deutschland wurden bisher keine Studien über den Gesundheitszustand von Überlebenden einer „coronavirus disease 2019“ (COVID-19) ein Jahr nach Entlassung aus der stationären Behandlung publiziert.

**Methoden:**

Diese retrospektive Kohortenstudie schloss alle Patienten mit Polymerase-Kettenreaktions(PCR)-Nachweis des „severe acute respiratory syndrome coronavirus 2“ (SARS-CoV-2) ein, die vom 15. März bis 31. Dezember 2020 in den internistischen Abteilungen des Klinikums Saarbrücken, einem Krankenhaus der Maximalversorgung, behandelt worden waren. Ein Telefoninterview mit den Überlebenden wurde frühestens 12 Monate nach stationärer Entlassung durchgeführt. Das Interview begann mit der offenen Frage, ob der Patient sich vollständig von COVID-19 erholt hatte. Im Falle der Angabe einer unvollständigen Erholung wurde der Patient gebeten, alle anhaltenden oder häufigen Beschwerden zu schildern, die er vor COVID-19 nicht gehabt hatte. Danach wurden – unabhängig von der Antwort auf die Eingangsfrage – geschlossene Fragen nach einem neuen Auftreten von anhaltender Müdigkeit, geistigen Problemen sowie Kopf- und Gliederschmerzen nach COVID-19 gestellt.

**Ergebnisse:**

Insgesamt 235 Überlebende wurden kontaktiert und 162 konnten in die Analyse eingeschlossen werden. In 55 von 162 Interviews (34,0 %) wurde mindestens ein anhaltendes COVID-19-Symptom („persistent COVID-19 symptom“ [PCS]) spontan berichtet. Vier von 55 Überlebenden mit PCS gaben 5 weitere PCS bei den geschlossenen Fragen an. Ein Überlebender, der die Eingangsfrage bejaht hatte, berichtete ein neu aufgetretenes PCS bei den geschlossenen Fragen. Anhaltende Müdigkeit (24,7 %), geistige Probleme (14,8 %), Atemnot (8,6 %), Gelenkschmerzen (6,8 %) und Kopfschmerzen (6,2 %) waren die häufigsten spontan berichteten PCS.

**Schlussfolgerung:**

Trotz einer Interviewtechnik, die darauf ausgerichtet war, eine Fehlattribution von Symptomen zu COVID-19 durch die Patienten zu vermeiden, berichtete ein Drittel der stationär behandelten Überlebenden über PCS 12 Monate nach stationärer Behandlung mit COVID-19.

Das komplette Beitrag ist in englischer Sprache abgefasst.

## Introduction

Severe acute respiratory syndrome coronavirus 2 (SARS-CoV-2) is responsible for the coronavirus disease 2019 (COVID-19) pandemic, which has resulted in a global healthcare crisis affecting millions of patients worldwide. In 2020 there were about 1,700,000 confirmed cases in Germany. Hospitalization occurred in 176,000 patients with 39,758 COVID-19-associated deaths reported for Germany. Mortality was increased by 2% in Germany due to COVID-19 in 2020 [18].

Whereas the initial focus was on the acute in-hospital treatment of patients, there are increasing reports of persistent COVID-19 symptoms (PCS) following the acute disease. Some politicians have raised the concern that the long-term health consequences of COVID-19 will become a relevant healthcare problem [1].

Different terms are used to describe the persistence of symptoms after viral clearance in patients affected by COVID-19 such as “post-acute COVID-19”, “ongoing symptomatic COVID-19”, “chronic COVID-19”, “post COVID-19 syndrome”, and “long-haul COVID-19” [10]. The World Health Organization launched the following definition of a post COVID-19 condition on October 8, 2021*: *“Post COVID-19 condition occurs in individuals with a history of probable or confirmed SARS-CoV‑2 infection, usually 3 months from the onset of COVID-19 with symptoms and that last for at least 2 months and cannot be explained by an alternative diagnosis. Common symptoms include fatigue, shortness of breath, cognitive dysfunction but also others and generally have an impact on everyday functioning. Symptoms may be new onset following initial recovery from an acute COVID-19 episode or persist from the initial illness. Symptoms may also fluctuate or relapse over time” [19].

More than 50 PCS have been described [12], even in patients asymptomatic at the time of the initial infection [4]. Results of prevalence studies of the most frequently reported PCS are influenced by the study design (e.g., prospective versus retrospective), the setting of the study (e.g., population-based or hospitalized cases), the time of assessment after the acute phase and the methods used to assess symptoms (structured interviews, standardized symptom questionnaires) [3]. PCS are more frequently reported by patients who had received inpatient hospital treatment compared to outpatients [12]. In a Chinese study of hospitalized patients, there was a decrease from 68% at 6 months to 49% at 12 months for report of at least one subsequent symptom [11]. Studies with 12-month follow-ups have been published for China [11], Italy [7], Switzerland [5], and Spain [13] but not for Germany.

Some PCS such as fatigue, sleep problems, and musculoskeletal pain are symptoms frequently reported by participants in general population surveys [6]. Patients who have survived COVID-19 may be influenced by media coverage of post COVID-19 sequelae. Therefore, framing a study to subjects as “Assessment of post-COVID-19 symptoms” can increase the risk that subjective symptoms, independent of SARS-CoV‑2, are falsely attributed to COVID-19.

The aim of the present study was to assess the prevalence, intensity and associated disability of PCS 12 months after detection of SARS-CoV‑2 in hospitalized patients in Germany using an interview technique suitable to reduce the risk of attribution bias by patients.

## Methods

### Study design and participants

This retrospective cohort study included all patients hospitalized with a positive diagnosis of SARS-CoV‑2 by swab PCR technique who were admitted between January 1 to December 31, 2020 to the two departments of internal medicine of the Klinikum Saarbrücken, a regional COVID-19 center and tertiary care hospital in Saarbrücken, Germany. We excluded all patients from the analysis of PCS if they (a) have been treated in other departments of the hospital because these departments have not perform a structured assessment of COVID-19 symptoms, (b) have died during the hospital stay or one week after discharge from the hospital or (c) could not be reached by telephone after five attempts or (d) declined to participate in the study.

All patients were tested for SARS-CoV‑2 by swab PCR technique on admission. Patients who were in the same room with another patient who had tested positive for SARS-CoV‑2 were tested. All patients who developed potential COVID-19 symptoms during the hospital stay despite a negative PCR test on admission were tested. All patients discharged to nursing homes or referred to other hospitals were tested for SARS-CoV‑2 by PCR technique 24 h before discharge.

All SARS-CoV-2-positive patients of both internal medicine departments were hospitalized in the infectious disease ward of the department of Internal Medicine 1, irrespectively of the disease leading to hospital admission, and/or the interdisciplinary intensive care unit (ICU). Patients were admitted to these wards for the following reasons:

- Patients with symptomatic COVID-19 from the emergency department;
- Patients with asymptomatic or symptomatic COVID-19, referred to the hospital for other diseases, but PCR confirmed SARS-CoV‑2 within 24 h after admission;
- Patients asymptomatic or symptomatic, referred to the hospital for other diseases, PCR SARS-CoV‑2 negative at admission, but became positive during the hospital stay (nosocomial COVID-19).

The study was approved by the ethical committee of the medical association of the Saarland (No. 108/21).

### Procedures

The medical history of all SARS-CoV-2-positive patients was assessed by a structured interview in the emergency department and/or in the infectious disease ward: Time since symptom onset and hospital admission or transfer to the infectious disease ward; symptoms (shortness of breath, fever, fatigue, limb pain, headache, sore throat, cough, rhinorrhea, altered smell, altered taste, nausea, diarrhea, vomiting). Clinical data during the hospital stay were retrieved from the electronic medical records, including demographic characteristics (age, sex, nursing home resident); clinical characteristics (body mass index and active medical comorbidities defined by medical history and medication at admission), laboratory test results (CRP, LDH) and treatment (medication: corticosteroids, antibiotics, antivirals, hydroxychloroquine; oxygen; ICU stay; noninvasive ventilation; mechanical ventilation, extracorporal membrane oxygenation). In patients who were asymptomatic at the time of admission to the infectious disease ward but became symptomatic during the hospital stay, the symptoms were assessed as described above.

Based on the clinical and radiology findings, COVID-19 infection was categorized as follows according to National Institutes of Health classification [15]:

- *Asymptomatic infection:* Individuals who test positive for SARS-CoV‑2 but who have no symptoms that are consistent with COVID-19.
- *Mild illness:* Individuals who have any of the various signs and symptoms of COVID-19 (e.g., fever, cough, sore throat, malaise, headache, muscle pain, nausea, vomiting, diarrhea, loss of taste and smell) but who do not have shortness of breath, dyspnea or abnormal chest imaging.
- *Moderate illness:* Individuals who show evidence of lower respiratory disease during clinical assessment or abnormal chest imaging and who have an oxygen saturation (SpO_2_) ≥ 94% on room air at sea level.
- *Severe illness: *Individuals who have SpO_2_ < 94% on room air at sea level (in case of chronic obstructive pulmonary disease we selected < 88% as threshold), a ratio of arterial partial pressure of oxygen to fraction of inspired oxygen (PaO_2_/FiO_2_) < 300 mm Hg, a respiratory rate > 30 breaths/min, or lung infiltrates > 50%.
- *Critical illness:* Individuals who have respiratory failure, septic shock, and/or multiple organ dysfunction.

### Interview

All telephone interviews except for one were conducted by one interviewer (WH) at least 12 months after hospital discharge. A single unilingual Arabic-speaking patient was interviewed by an Arabic speaking physician (DEF) adhering to the interview guideline. The contact details of the patients and/or their relatives and/or nursing home was retrieved from the medical charts. The interviewer introduced himself as a consultant of the department of Internal Medicine 1, Klinikum Saarbrücken and the reason for the call was explained as follows: With the approval of the Medical Association of the Saarland, the Klinikum Saarbrücken is conducting a study of the current health status of patients who had received inpatient medical treatment one year before and had tested positive for the coronavirus. Patients were informed that participation in the study was voluntary and the laws of data protection were respected. After an informal consent to participate, patients were asked the following: “Did you recover completely from your COVID-19?” If the patient answered no, he/she was asked: “Please tell me about your ongoing symptoms”. For each reported symptom, the patient was asked: “Please estimate the intensity of your symptoms (slight, moderate, severe or very severe) and the degree of impairment associated with these symptoms in your daily life (slight, moderate, severe or very severe)”. If the patient reported impairing symptoms, he/she was asked if they were on sick leave because of the symptoms (if employed). Thereafter, the patient was asked if he/she had experienced the symptoms before COVID-19. If yes, the symptom was not classified as PCS. A symptom was classified as PCS if the patient reported that the symptoms had persisted after discharge or started within 4 weeks after the positive test and had never been experienced before. In addition, spontaneous report of worsening of pre-existing diseases was recorded, but without prompting. Finally, closed questions addressing potential ongoing symptoms that had not occurred before COVID-19 (if not reported spontaneously in the open-ended format) were as follows: frequent or permanent headaches, pain in the muscles or joints, physical fatigue or rapid exhaustion, cognitive dysfunction (problems with concentration or memory)—independently of the subjective status of recovery. If the patient affirmed a symptom, he/she was asked to rate the intensity and disability as outlined above. These symptoms were selected because of our special interest in post-COVID-19 pain and fatigue [8]. We limited the interview to the above format, without addition of other potential PCS questions or questionnaires so as to not overburden the patients. If a patient was unable to answer questions independently, we used the same interview technique to question a proxy or a close contact of the patient (e.g., partner, adult child, residency home staff). In the event of death after hospital discharge, we questioned whether death was COVID-19 related or not. At the end of the interview on PCS the interview partners were invited to ask questions about the study.

Written information about the study including the modalities of data protection and a consent form was sent to the interview partners in a stamped self-addressed envelope after the telephone interview. They receivers were asked to send the signed consent form back to the senior author the study (WH).

### Statistical analyses

Descriptive statistics, i.e., frequencies as well as means and standard deviations were used to present characteristics of the study population. Group comparisons were conducted by Χ^2^ test for categorical variables and by Mann–Whitney test for continuous variables. *P*-value is reported without defining a so-called level of statistical significance.

## Results

### Study sample

**Fig. 1:**
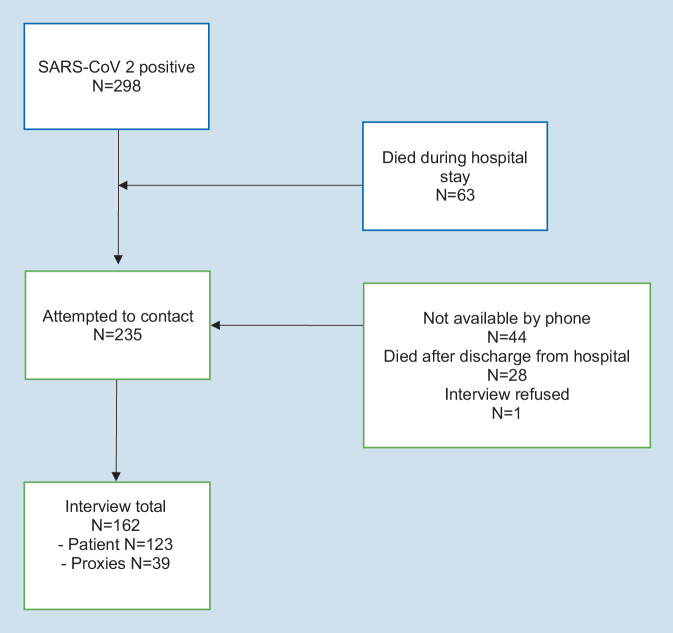
Flow diagram of the study population

Between March 15 and December 31, 2021, SARS-CoV‑2 was detected by nasal PCR in 482 of 25,228 (1.9%) of all inpatients of the Klinikum Saarbrücken. Of the 482 (61.8%) SARS-CoV-2 patients, 298 were managed in the internal medicine departments. Of the 298 patients, 170 (57.0%) were admitted because of COVID-19 symptoms, 68 (22.8%) were admitted because of other diseases but tested positive for SARS-CoV‑2 at admission and 60 (20.1%) tested positive for SARS-CoV‑2 during the hospital stay (nosocomial COVID-19). Figure 1 shows the flowchart of patients according to study inclusion. Sixty-three patients (21.1%) died during the hospital admission and 30 patients (10.1%) died after hospital discharge and before the end of follow-up. Cause of death in 29 of the 30 patients after discharge was unrelated to COVID-19 according to report of residency home nurses or relatives, and one person committed suicide as he could not tolerate the loss of smell and taste after COVID-19. Forty-four survivors (14.8%) could not be reached by telephone despite five attempts. One patient (0.3%) did not agree to participate. The final sample therefore consisted of 162/482 (33.6%) patients.

A personal telephone interview was conducted in 123 (76.0%) survivors, and a family member or nursing home staff was interviewed in 33 (24%) survivors who were unable to respond personally due to dementia or aphasia after a stroke. All proxy respondents had a personal knowledge of the patient prior to COVID-19. At least one ongoing symptom was spontaneously reported for 55 (34%) of 162 subjects. Four of 55 survivors with PCS reported five additional symptoms, but only in response to the closed questions (three for physical fatigue, one for cognitive dysfunction, one for headache). One survivor who responded to the open-ended question that he was completely recovered, reported new onset cognitive dysfunction in response to the closed questions. Taken together, the prevalence of spontaneously reported versus closed question reported PCS was 34.0%. The prevalence of PCS increased by 0.6% after closed questions. Report of PCS was as follows: 25 (44.6%) survivors reported one, 15 (26.8%) reported two, 9 (16.1%) reported three and 7 (12.5%) reported > 3 PCS. The most frequent new onset and spontaneously reported PCS were physical fatigue (24.7%), cognitive dysfunction (14.8%), shortness of breath (8.6%), pain in muscles and joints (6.8%) and headache (6.2%). Muscle and joint pain and headache as only PCS were not reported. Of the 11 patients with muscle and joint pain, 7 (63.6%) reported headache, 9 (81.8%) physical fatigue and 7 (63.6%) cognitive dysfunction. Of the 10 patients with headache, 7 (70%) reported muscle and joint pain, 10 (100%) physical fatigue and 6 (60%) cognitive dysfunction. The intensity of symptoms and associated impairment ranged from slight to very severe (Table 1). Four of the 66 (6.1%) survivors who were working full-time before acute COVID-19 were still on sick leave due to PCS.

**Table 1:** Prevalence, intensity and associated disability of spontaneously reported new onset persistent symptoms related to coronavirus disease 2019 (COVID-19) in 162 survivors at one year after hospital discharge (order according to frequency)

Symptom	Prevalence *N *(%)	Intensity (1 = slight … 4 = severe) Mean (SD)	Disability (0 = none; 4 = very severe) Mean (SD)
Fatigue	40 (24.7)	2.3 (0.9)	2.3 (0.9)
Cognitive dysfunction	24 (14.8)	2.4 (1.4)	2.2 (1.2)
Shortness of breath	14 (8.6)	1.9 (0.6)	1.6 (1.1)
Pain in muscles and joints	11 (6.8)	2.3 (0.8)	2.0 (1.1)
Headache	10 (6.2)	2.4 (0.7)	1.3 (1.2)
Cough	3 (1.8)	2.3 (0.6)	1.3 (1.2)
Altered smell/taste	3 (1.8)	1.7 (1.2)	0.3 (0.6)
Posttraumatic stress symptoms	2 (1.2)	3.0 (1.0)	3.0 (1.0)
Sleep problems	2 (1.2)	3.0	1.5 (1.5)
Anxiety	1 (0.6)	3.0	3.0
Depression	1 (0.6)	3.0	3.0
Disturbance of sensitivity in one leg	1 (0.6)	1.0	1.0
Loss of appetite and weight	1 (0.6)	2.0	2.0
Nausea	1 (0.6)	3.0	1.0
Pain in hands and feet	1 (0.6)	3.0	3.0
Pruritus	1 (0.6)	2.0	2.0
Thoracic burning	1 (0.6)	2.0	2.0
Vertigo	1 (0.6)	1.0	2.0
Weakness of forefoot	1 (0.6)	1.0	1.0

*SD* standard deviation

Three patients reported worsening of pre-existing pulmonary disease and 2 patients had worsening of pre-existent fibromyalgia syndrome. One patient had a new onset of type 2 diabetes during the hospital stay. Rapid worsening of dementia was reported by proxy interview for 5 survivors.

There were some signals that survivors with PCS had a more severe course of disease (CRP at admission, severity of COVID-19, length of hospital stay) than survivors without PCS. There were no age differences between the two groups, but the proportion of nursing home residents was higher in the non-PCS group (Table 2).

**Table 2:** Comparison of demographic and clinical baseline characteristics of inpatient survivors with and without persistent symptoms of COVID-19 one year after hospital discharge

	Persistent symptoms related to COVID-19 *N* = 56	No persistent symptoms related to COVID-19 *N* = 106	*p*-value
**Gender**			
*Female, N* (%)	25 (44.6)	49 (46.2)	0.89
**Age**, Mean (SD)	62.2 (16.9)	66.6 (17.2)	0.12
**Age group**			
*<* *20 years (%)*	1 (1.8)	0	0.15
*20–39 years (%)*	2 (3.6)	8 (5.1)	
*40–59 years (%)*	18 (32.1)	26 (46.4)	
*60–79 years (%)*	25 (44.6)	42 (26.9)	
*>* *80 years (%)*	10 (17.8)	29 (18.6)	
**Nursing home resident**, *N* (%)	5 (8.9)	21 (19.8)	0.0004
**German place of residence**, *N* (%)	101(95.3)	54 (96.4)	0.94
**Working**, *N* (%)	26 (46.4)	40 (37.7)	0.31
**Number of active comorbidities**, *N* (%)	1.6 (1.3)	2.0 (1.6)	0.06
**Number of COVID-19 symptoms at admission**, Mean (SD)	3.5 (2.2)	2.2 (2.2)	0.17
**CRP at the time of admission to COVID-19 ward (mg %; normal <** **5** **mg%)**	63.0 (63.2)	43.3 (55.5)	0.05
**LDH at the time of admission to COVID-19 ward (U/l); normal <** **220** **U/l**	324 (129)	281 (112)	0.08
**COVID-19 severity**			
*Asymptomatic*	7 (12.5)	40 (37.7)	0.02
*Mild*	16 (28.6)	22 (20.8)	
*Moderate*	16 (28.6)	24 (22.6)	
*Severe*	14 (25.0)	19 (17.9)	
*Critical*	2 (3.6+)	1 (0.9)	
*Critical (Multiorgan failure)*	1 (1.8)	0	
**Management**			
*Oxygen in ward*	30 (53.8)	36 (34.0)	0.02
*Noninvasive ventilation in ICU*	2 (3.6)	5 (4.7)	0.44
*Invasive ventilation in ICU*	3 (5.4)	1 (0.9)	0.09
*ECMO*	1 (1.8)	0	–
**Length of hospital stay in days, **Mean (SD)	10.4 (10.8)	9.2 (8.2)	0.02

## Discussion

### Summary of main results

In this single center retrospective study of 162 SARS-CoV‑2 convalescent patients, we aimed to reduce attribution bias by initiating the telephone interview with an open-ended question about current health status, followed by closed questions about symptoms that had not been present prior to the acute SARS-CoV‑2 infection. In 55 of 162 interviews (34.0%) patients spontaneously reported of at least one new onset symptom. Physical fatigue (24.7%), cognitive dysfunction (14.8%), shortness of breath (8.6%), pain in muscles and joints (6.8%) and headache (6.2%) were the most frequently reported PCS. Symptoms-related disability was rated mostly as moderate.

### Comparison with other studies

The different settings (inpatient versus outpatient), the differences in the severity of acute COVID-19 and the different methods of assessment (structured interviews, symptom questionnaires, open questions) does not allow a direct comparison with other studies that have reported on at least a 1-year follow-up.

Remarkably, the most frequently reported PCS in this study were the same as those explicitly mentioned by WHO in the post COVID-19 definition, namely fatigue, cognitive dysfunction and shortness of breath [19].

Huang et al. assessed 1276 Chinese COVID-19 survivors 1 year after hospitalization in a single hospital from January 7 to May 29, 2020 (75% with moderate to severe COVID-19) with a response rate of 53%. Forty nine percent reported at least one PCS. The most frequently reported symptoms assessed by interview and questionnaires were anxiety/depression (26%), fatigue (muscle weakness; 20%), sleep problems (17%) and joint pain (12%). Most survivors had a good physical and functional recovery over time, and had returned to their original work and life, but current health status was still lower than for the control population. Lung diffusion impairment and radiographic abnormalities remained common at 12 months for those who had been critically ill [11].

A study from Switzerland included 90 of 301 adult patients hospitalized for confirmed COVID-19 in two Swiss tertiary-care hospitals between March and June 2020 (inclusion rate 30%): 55% had received oxygen by nasal canula or noninvasive ventilation and 11% underwent mechanical ventilation. Symptoms assessed by interview and questionnaires were reported by 70% one year after hospitalization and included fatigue (46%), concentration difficulties (31%), shortness of breath (21%) and post-exertion malaise (20%). Thirty-four (38%) patients indicated that their symptoms limited their quality of life. A total of 34% of patients reported one or two symptoms, and 36% reported ≥ 3 symptoms after 1 year [5].

In a Spanish single center study, COVID-19 patients who were either hospitalized or discharged from the emergency room from 1 March to 1 June, 2020 were assessed 1 year after discharge by questionnaire. Of the 321 patients with mild-to-moderate COVID-19 discharged directly from the emergency room, and 445 with severe-to-critical illness admitted to hospital, potential post-COVID-19 symptoms were assessed in 543 patients. Any clinical complaint was reported by 90.1% of patients who were hospitalized and 80.4% of those discharged from the emergency room. Ongoing symptoms attributed to COVID-19 were reported by 66.8% and 49.5% of patients, respectively. Breathlessness (41.6%), tiredness (35.4%), ageusia (30.2%), and anosmia (26.3%) were the most common complaints [13].

An Italian study consecutively assessed 304 outpatients with mild-to-moderate COVID-19 between March 1 and March 31, 2020 (inclusion rate 86%). Persistence of at least one symptom at 12 months was reported by 161 patients (53.0%) with reports of feeling tired (27.3%), smell or taste impairment (22.0%), shortness of breath (12.8%), and muscle pain (9.2%) [7].

A systematic review of 27 peer-reviewed studies and 6 preprints found an overall prevalence of post-COVID myalgia, joint pain, and chest pain ranged from 5.65–18.15%, 4.6–12.1%, and 7.8–23.6%, respectively, at different follow-up periods during the first year post infection [12]. The proportion of patients with de novo chronic musculoskeletal pain in our study was within the range reported by a systematic review [9].

A few patients in our study spontaneously reported psychological symptoms such as posttraumatic stress disorder (PTSD) symptoms (1.2%) and anxiety/depression (1.2%). Using symptom-based questionnaires in the Swiss study, the prevalence of anxiety and depressive symptoms was higher with 13% and similar for PTSD with 3% [5].

In line with other studies, the prevalence of PCS increased with disease severity [5, 11].

Other long-term studies have documented newly diagnosed asthma, chronic obstructive pulmonary disease (COPD) and diabetes in a very small number of patients (1% to 2%), the need for intensification of COPD treatment, and increased cognitive impairment in patients with previous Alzheimer’s disease or other acquired cognitive impairment or dementia, too [13]. An association of COVID-19-induced anosmia and ageusia with depression and suicidal ideation has also been reported by another study [21].

The majority of survivors in this study reported only slight to moderate associated disability. However, four survivors were still on sick leave. Another German study found that 11% of patients could not fully participate in everyday and work life 7 months following mild SARS-CoV‑2 infection [4].

### Attribution bias

Remarkably, all long-term studies with a follow-up of 1 year did not report on the framing of the study to the participants, i.e., which study aims were disclosed. In addition, no study discussed the risk of an attribution bias that could result in a false positive rate of PCS. Attribution bias was largely excluded in our present study by the introductory “framing” by using an open-ended question, and by the very low number of symptoms reported when participants were asked closed questions.

### Not all symptoms after acute COVID-19 are due to SARS-CoV-2

Symptoms reported after acute COVID-19 can be categorized as follows:

*Somatic sequelae of acute COVID-19*, e.g., persistent dyspnea by impaired pulmonary function [20] in patients with previously healthy lungs or loss of smell/taste that can persist for an undetermined period of time.

*Somatic sequelae of COVID-19 therapy*, e.g., musculoskeletal pain by prolonged bedrest. Lockdown with reduced physical activity and increase of body weight might contribute to musculoskeletal pain.

*Psychological sequelae of COVID-19 therapy*, e.g., PTSD after ICU treatment.

*Biopsychosocial sequelae of acute COVID-19, its therapy and its social consequences*: Some somatic symptoms such as fatigue and cognitive disturbances can be explained by an interaction of biological and psychological variables, e.g., by inflammatory response during acute COVID-19, pre-existing depression and psychological distress by social distancing and lockdowns [2]. We can give narrative support for the biopsychosocial model. Two nurses with PCS after moderate/severe COVID-19 reported worsening of fatigue and cognitive disturbances following conflict with the workers compensation board about acknowledging PCS as an occupational illness. In defining the post-COVID-19 condition as symptoms that cannot be explained by an alternative diagnosis, the WHO fails to capture a critical biopsychosocial component of illness.

*Symptoms erroneously attributed to COVID-19*: Symptoms can be explained by previous (undiagnosed) or new (undiagnosed) diseases after acute COVID-19. The risk to falsely attribute symptoms which are common in the general population such as fatigue and musculoskeletal pain is highlighted by a large French population-based study of 26,823 individuals tested for anti-SARS-CoV‑2 antibodies by an enzyme-linked immunosorbent assay between May and November 2020. Between December 2020 and January 2021, participants reported whether they believed they had experienced COVID-19 infection and whether they experienced any physical symptoms during the previous 4 weeks, and if there were physical symptoms whether they had persisted for at least 8 weeks. Persistent physical symptoms were more associated with the belief of having been infected with SARS-CoV‑2 than with having a laboratory-confirmed infection [14].

### Strengths and limitations

The framing of the study aims to participants and the interview technique reduced the risk of an attribution bias. We also did not exclude patients with dementia and non-German speaking patients.

We may have underestimated the prevalence of PCS because we relied mainly on open-ended questions rather than symptom lists and questionnaires. There is higher reporting of adverse events in placebo-controlled medication trials when symptom lists rather than open-ended questions are administered [17]. We hypothesize that this likely applies to PCS studies.

Ideally, a control group of people reporting de novo symptoms since the COVID-19 pandemic, but without COVID-19, would have allowed an estimation of the amount of attribution bias to PCS.

Although outpatients were not included in the present study, our findings are more aligned with the outpatient cohort of Augustin et al. [4] than the inpatient cohorts with 1‑year follow-up in China [11], Spain [13] and Switzerland [5].

The study design was retrospective and COVID-19 symptoms leading to hospital admission were not regularly monitored after discharge. Furthermore, the study design did not include medical examination, or neurocognitive tests and we did not systematically assess for pre-existing disease which might explain some PCS. Therefore, we are unable to confirm which PCS could be attributed to pre-existing diseases, to COVID-19, to undiagnosed new diseases after discharge independent of COVID-19, or to somatic and psychological sequelae of COVID-19 treatment and lockdown arrangements.

The proxy response for patients not able to be personally interviewed may have been inaccurate regarding PCS.

Laboratory tests (CRP, LDH) might not have accurately reflected the COVID-19 associated inflammatory response due to the presence of some other comorbid condition.

The study size is rather small and statistical comparisons are underpowered.

## Perspectives

The German Network University Medicine (NUM) within the National Pandemic Cohort Network (NAPKON) continues to monitor adults after COVID-19 with regards to secondary diseases and health-related quality of life (NAPKON-POP/COVIDOM study). There is a special interest group for chronic pain [16].

## Conclusions for clinical practice

- The consequences of COVID-19 can be diverse and prolonged. Our results suggest that most patients will experience a self-limited acute infection with full recovery, but every third patient develops symptoms that persist for at least 1 year. For some COVID-19 survivors, persisting symptoms are sufficiently severe to preclude return to employment.
- New-onset headache and pain in muscles and joints are frequently associated with each other and with physical fatigue and cognitive disturbances.
- The severity of acute COVID-19 might increase the risk of post-COVID-19 conditions.
- Some post-COVID-19 symptoms such as shortness of breath can be explained by persistent structural changes of the pulmonary systems.
- The most frequently reported PCS, namely fatigue and cognitive disturbances, are common symptoms in the general population and should not be solely attributed to infection by SARS-CoV‑2 virus.
- Acute COVID-19 can worsen pre-existing diseases, e.g., chronic obstructive pulmonary disease (COPD), dementia or FMS.
- Pain medicine physicians should be involved in the management of chronic pain (headache, musculoskeletal system) and mental health care specialist in the management of fatigue and cognitive problems after COVID-19.

## Declarations

For this article no studies with human participants or animals were performed by any of the authors. All studies performed were in accordance with the ethical standards indicated in each case.
